# Evaluation of the Effectiveness of Pregabalin in Alleviating Pain Associated with Fibromyalgia: Using Functional Magnetic Resonance Imaging Study

**DOI:** 10.1371/journal.pone.0074099

**Published:** 2013-09-06

**Authors:** Seong-Ho Kim, Youngho Lee, Sunggun Lee, Chi-Woong Mun

**Affiliations:** 1 Division of Rheumatology, Department of Internal Medicine, Haeundae Paik Hospital, College of Medicine, Inje University, Haeundae-gu, Busan, South Korea; 2 Department of Biomedical Engineering/UHRC, Inje University, Gimhae, Gyeongnam, South Korea; Charité University Medicine Berlin, Germany

## Abstract

**Purpose:**

To assess the efficacy of pregabalin by showing differences in the neuronal activities of fibromyalgia (FM) patients before and after longitudinal treatment using functional magnetic resonance imaging (fMRI).

**Materials and Methods:**

In total, 21 female patients with FM and 11 age- and gender-matched healthy controls participated. FM patients underwent fMRI at baseline and following pharmacological therapy with pregabalin to diminish their pain. Pressure-pain stimuli were delivered on the subject’s thumbnail bed during fMRI scans. Brain activation regions in fMRI were evaluated for longitudinal changes using a paired *t*-test. Changes in clinical features were also assessed with the Fibromyalgia Impact Questionnaire (FIQ), Brief Fatigue Inventory (BFI), Beck Depression Inventory (BDI), Widespread Pain Index (WPI), Symptom Severity Scale Score (SSS), and State-Trait Anxiety Inventory (STAI).

**Results:**

Clinical scores were reduced significantly following therapy with five of the six clinical tests (FIQ, BFI, BDI, WPI, SSS; *p* < 0.05). Brain activation post-treatment was significantly lower than that pre-treatment in 13 regions of the brain (*p* < 0.001).

**Conclusions:**

Our findings confirm that pregabalin influences aspects of the whole pain matrix, using fMRI, inducing longitudinal changes in neuronal activity during the pain state, and that it reduces pain and other core symptoms of FM. This method could be applied to other longitudinal clinical trials of pharmacological treatments for FM.

## Introduction

Fibromyalgia (FM) is characterized by chronic widespread musculoskeletal pain and allodynia [1]. Other symptoms include weakening fatigue, sleep disturbances/non-restorative sleep, and cognitive impairment [2]. Several studies have shown that FM patients and healthy controls detect the same levels of stimuli; however, investigation of sensitivity to experimentally induced pain has shown that patients with FM have lower pain thresholds and higher pain ratings in response to pressure, heat, cold, and electrical stimuli [3–5]. The etiology of FM remains unknown, and no consistent underlying mechanism has been identified. In several hypotheses, however, FM patients have a lower pain threshold because of their higher sensitivity to pain stimulation [6].

It is well known that functional magnetic resonance imaging (fMRI) is an invaluable tool for neuroscientific research because it provides a functional view of the brain at the system level [7]. Stimulation related to neuronal activation results in increased regional cerebral blood flow (rCBF) to meet increased metabolic demands [3,5,7]. Several previous studies have demonstrated abnormal pain processes in FM patients using fMRI [3,8]. Gracely et al. [3] reported that comparable levels of subjectively reported painful stimulation resulted in similar patterns of brain activation in both FM patients and healthy controls, whereas, for similar intensities of pressure pain, there was no common activation region but greater effects in specific pain-processing regions. These regions were the sensory-discriminative components of the brain, such as the primary (SI) and secondary somatosensory cortex (SII), as well as the affective-motivational components, such as the insula and anterior cingulate cortex (ACC).

Presently, treatment of FM is symptom-based, seeking to alleviate pain, increase restorative sleep, and enhance physical and social functioning [9]. Pharmacological treatments include medications that have a modulatory function, such as tricyclics, selective serotonin reuptake inhibitors, and serotonin/norepinephrine reuptake inhibitors [10]. Pregabalin (PGB) is a structural analog of the neurotransmitter γ-aminobutyric acid (GABA). Pregabalin binds to the α_2_-δ (alpha_2_-delta) subunit of the voltage-dependent calcium channel in the central nervous system (CNS) and decreases the release of neurotransmitters, such as glutamate, noradrenaline, and substance P [11]. This mechanism is assumed to be the basis for the analgesic, anticonvulsant, and anxiolytic effects of the drug [12]. According to Crofford et al., pregabalin reduced pain and other core symptoms of FM, including improving fatigue and sleep disturbances [9]. Thus, it could be that pregabalin induces longitudinal changes in neuronal activity in the pain state.

We hypothesized that the clinical improvements in the pain state of FM patients were related to the effects of the medication, pregabalin, in the central nervous system. The fMRI technique was used to characterize the pattern of increased brain activation produced when subjective pressure-pain stimulation was applied to the thumbnail bed of FM patients and healthy control subjects. These patterns of brain activation were compared before and after pregabalin treatment.

## Methods

### Subjects

**Figure 1:**
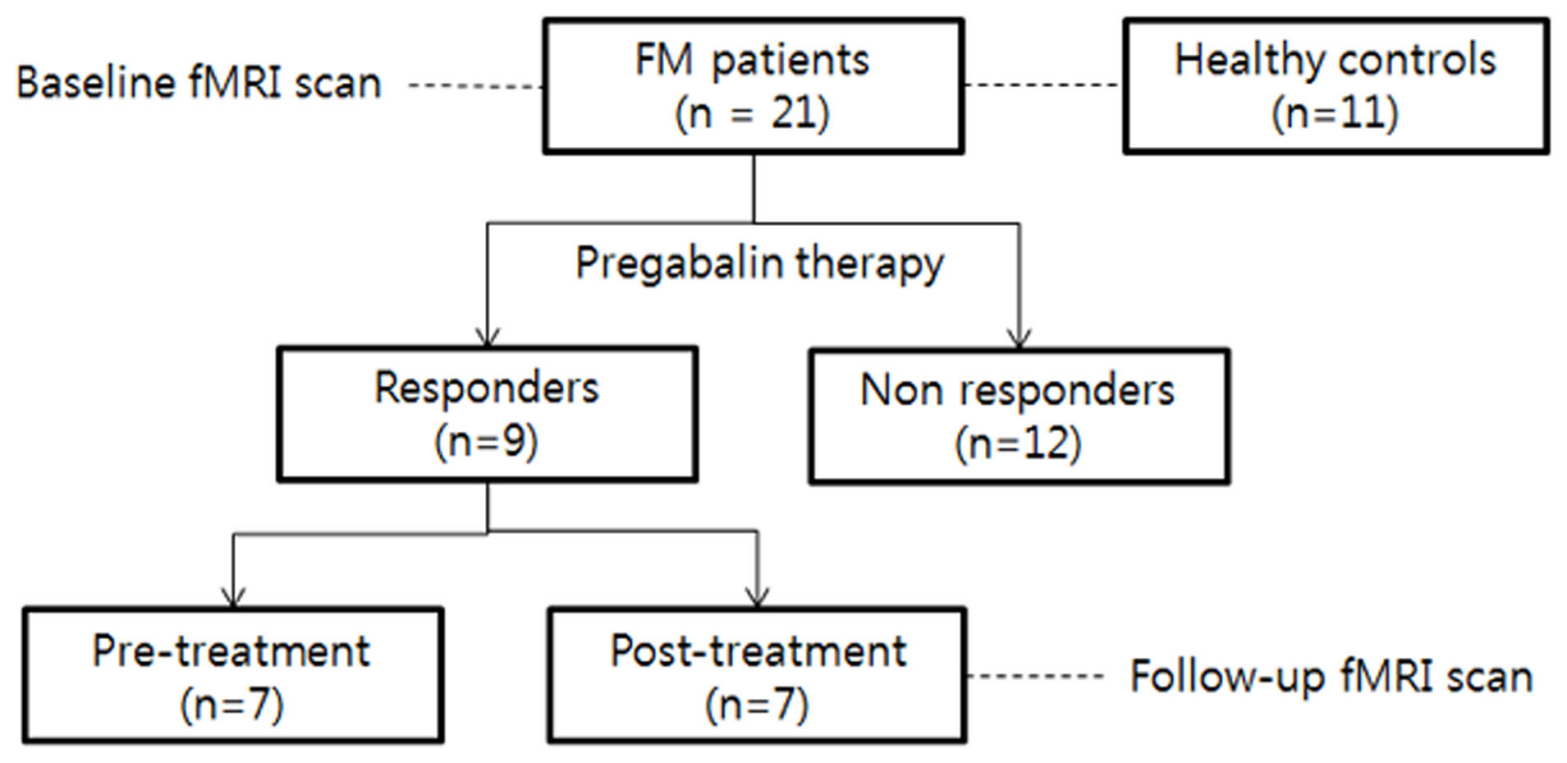
Outline of the study design and classification of subjects used for evaluation of the effectiveness of pregabalin in the treatment of patients with fibromyalgia, using fMRI.

In this study, 21 female patients (51.3±8.4 years of age; range, 24-63) with FM and 11 age- and gender-matched (46.5±12.0; range 24-62) healthy controls participated. Patients underwent routine clinical treatment using PGB. Patients were divided into two groups, responders and non-responders, according to decreases in their visual analog scale (VAS) scores for pain of above 50% after the treatment. Nine patients responded to the drug and were considered ‘responders,’ whereas 12 patients did not, ‘non-responders.’ Figure 1 shows the classification of the subjects who participated. All FM patients underwent baseline fMRI before pregabalin medication and only responders underwent follow-up fMRI scans. In the responder group, only seven of nine patients underwent MRI scans after PGB treatment. All patients were evaluated using several clinical tests: the Fibromyalgia Impact Questionnaire (FIQ), Brief Fatigue Inventory (BFI), Beck Depression Inventory (BDI), Widespread Pain Index (WPI), Symptom Severity Scale Score (SSS), and State-Trait Anxiety Inventory (STAI) 1 and STAI2 scales.

### Ethics Statement

The study protocol was approved by the Institutional Review Board at Inje University Haeundae Paik Hospital (No. 2011-009). All participants agreed to participate in the study and provided written informed consent.

### Pain stimulation

Pressure-pain stimuli were delivered using a specially designed hydraulic device capable of transmitting controlled pressure to a surface placed on the subject’s thumbnail bed. As in other studies [13], a hydraulic piston was connected via a combination of valves to a second piston, which produced controlled and repeatable stimuli that approached a rectangular waveform. In a pre-fMRI baseline session, the pressure-pain sensitivity of the subjects was evaluated using a numerical analog descriptor scale of pain intensity, a subjective scaling of suprathreshold sensations. Pressure-pain sensations were evoked by an ascending series of discrete stimuli; the initial stimulation pressure was 0.35 kg/cm^2^ and then increased until either the subject’s level of pain tolerance or a maximum pressure of 2.81 kg/cm^2^ was reached. Following the ascending series, eight pressure-pain stimuli (intensities of 0.35, 0.70, 1.05, 1.40, 1.75, 2.1, 2.46, and 2.81 kg/cm^2^) were delivered during 5 s and each subjective pain intensity was recorded on a Gracely Box Scale (GBS) sheet [14] to determine strong pain ratings (14 among 21 levels).

**Figure 2:**
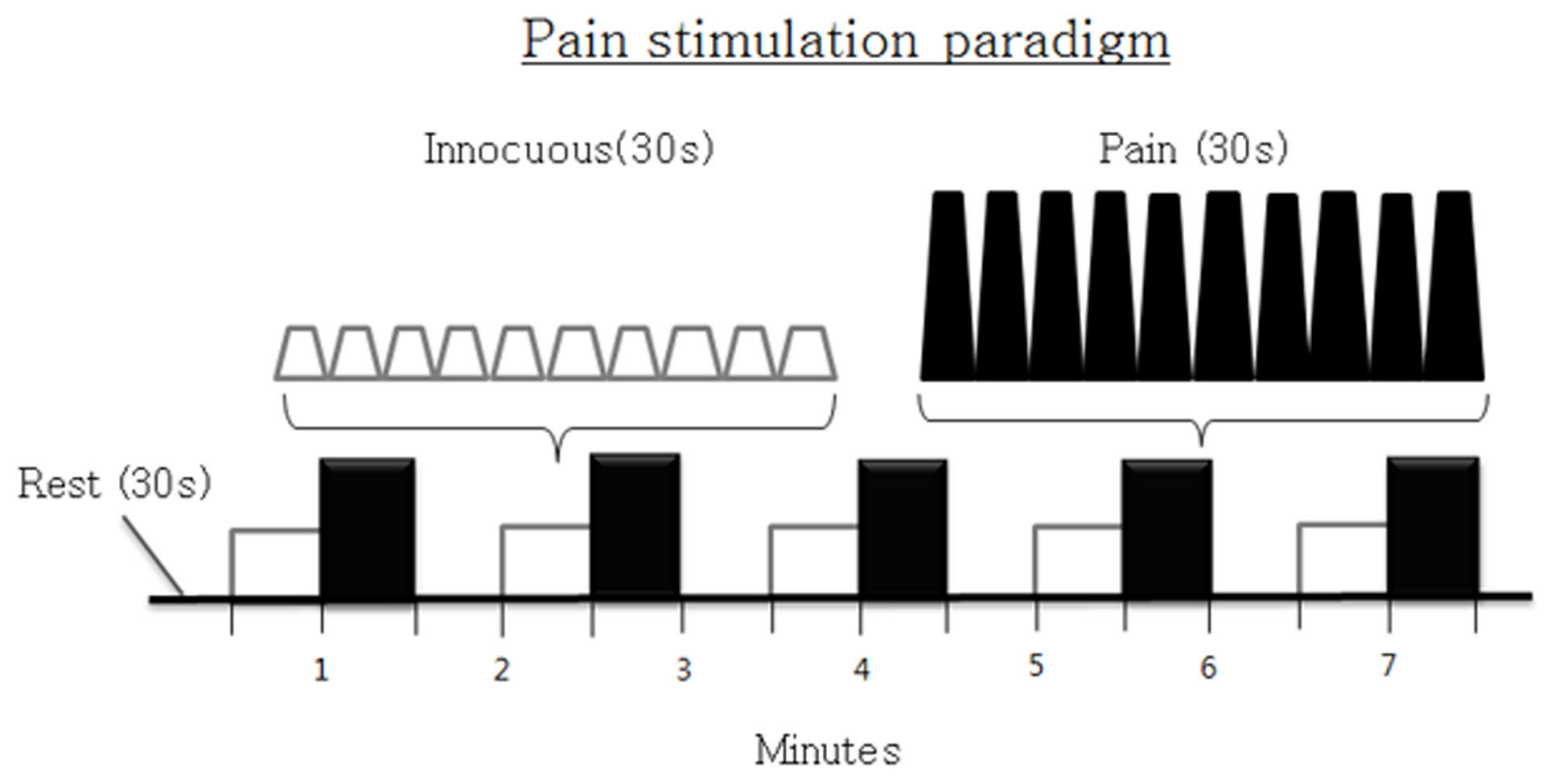
Pain-stimulation paradigm of the fMRI scan.

Figure 2 shows the pain-stimulation paradigm for the fMRI scan. One cycle of this paradigm consisted of three sessions: rest and two pain-stimulus periods, such as allodynia (innocuous) and noxious (strong, level 14 on the GBS). All three sessions were designed to have durations of 30 s, so that the length of one cycle was 1 min 30 s, and two stimulus sessions consisted of 10 consecutive pressure pulses with a width of 3 s. This paradigm was synchronized to the fMRI scan with TR of 3 s so that 10 functional images were collected during the 30-s stimulus. The cycle was repeated five times through the paradigm for a total scan time of 7 min 30 s for 150 volumes.

### MRI acquisition

MRI scans were conducted using a 3-T whole body clinical scanner (Philips, Achieva, Netherlands) with a 32-channel SENSE (SENSitivity Encoding) head coil. Three-dimensional T1-weighted MRI scans using a turbo field echo (TFE) sequence (TR/TE = 9.9/4.6 ms, flip angle = 8°, matrix size = 240 × 240, field of view (FOV) = 240 mm, slice thickness = 1 mm) were performed for anatomical information, followed by two functional MR scans using multi slice echo planar imaging (EPI) acquisition (TR/TE = 3000/30 ms, flip angle = 65°, matrix size = 220 × 220, FOV = 128 mm, slice thickness = 4 mm).

### Image analysis

Imaging data were analyzed with Statistical Parametric Mapping 8 (SPM 8; Wellcome Department of Imaging Neuroscience, London) implemented in MATLAB 2010 (Mathworks Inc, Natick, MA). Motion artifacts in functional images were corrected throughout the processing of realignments, coregistering, and smoothing. Spatial normalization was conducted using a 12-parameter affine transformation to match each image volume to the template-volume image by minimizing the residual sum of squared differences between the image and the template using 152 Montreal Neurological Institute (MNI) template images as a standard space model. The spatially normalized images were smoothed with a 6-mm FWHM isotropic Gaussian kernel to improve the SNR. After regression analysis of preprocessed fMRI data, using a hemodynamic response function (HRF), correlation verification was conducted. Statistical analysis to evaluate the brain activation in each group was carried out at the subjective pain intensity. Clusters were defined as a volume of activations with more than 50 statistically significant voxels. Significant activations according to the stimulation were assessed by one sample t-test. In group analysis, two-sample t-test was used to compare the brain activation between FM patients and healthy control (corrected p < 0.05), responder and non-responder (uncorrected p < 0.01). Additionally, comparison between pre- and post-treatment was performed using paired t-test (uncorrected p < 0.01)

### Statistical analysis

The analysis was conducted using the Statistical Program for the Social Sciences version 12.0 (SPSS Inc., Chicago, IL, USA). To compare clinical scores and pain threshold between the groups t-test for independent groups was used, and between pre- and post-treatment Wilcoxon signed rank test was used.

## Results

### Pressure-pain threshold

For the pressure-pain stimulation delivered on the left thumbnail bed, the FM patient group had significantly lower pressure-pain thresholds than the healthy control group (pressure pain: 1.67±0.28 *vs.* 2.46±0.02; *p* < 0.01). Furthermore, the pre-treatment group showed significantly higher pressure-pain sensitivity than the post-treatment group (pressure pain: 1.78±0.14 *vs.* 2.60±0.04; *p* < 0.01).

### Clinical tests

**Figure 3:**
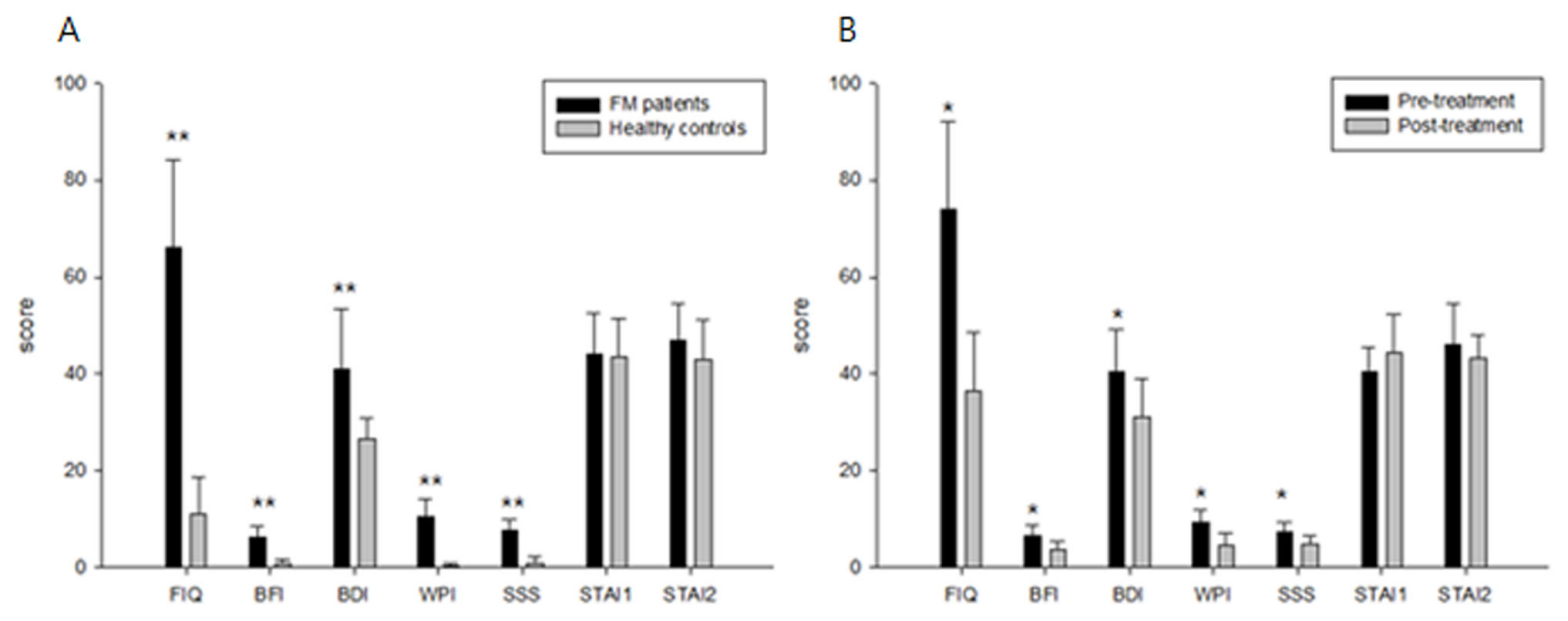
Statistical comparisons of clinical scores. (Mean and SD.) (A) FM patients and healthy controls (B) pre-treatment group and post-treatment group. Fibromyalgia Impact Questionnaire (FIQ), Brief Fatigue Inventory (BFI), Beck Depression Inventory (BDI), Widespread Pain Index (WPI), Symptom Severity Scale score (SSS), and State-Trait Anxiety Inventory (STAI). * *p* < 0.05, ** *p* < 0.01.

As shown in Figure 3 (A), clinical scores (mean±SD) of FM patient group were 65.98±18.11 (FIQ), 6.34±2.13 (BFI), 41.10±12.17 (BDI), 10.43±3.67 (WPI), 7.61±2.29 (SSS), 44.10±8.46 (STAI1) and 47.05±7.58 (STAI2). While the corresponding clinical scores of healthy control group were 10.97±7.65, 0.82±0.85, 0.18±0.6, 0.82±1.47, 43.64±7.90 and 42.91±8.14. Clinical scores of both groups were significantly different in terms of FIQ, BFI, BDI, WPI, and SSS (all *p* < 0.001). However, there was no significant difference in STAI1 or STAI2 scores. In Figure 3 (B), post-treatment scores on five clinical tests (FIQ: 36.4±12.08, BFI: 3.63±1.86, BDI: 31.17±7.88, WPI: 4.6±2.61, SSS: 4.8±1.79) showed significant reductions in clinical features (*p* < 0.05) *versus* those pre-treatment (FIQ: 73.87±18.26, BFI: 6.63±2.23, BDI: 40.50±8.8, WPI: 9.4±2.61, SSS: 7.4±1.95). FIQ and WPI scores demonstrated up to a twofold change and BDI scores showed the least difference, a 1.3-fold change. In contrast, STAI1 and STAI2 were slightly, but not statistically significantly, changed.

### Comparison of brain activation between FM patients and healthy controls

**Figure 4:**
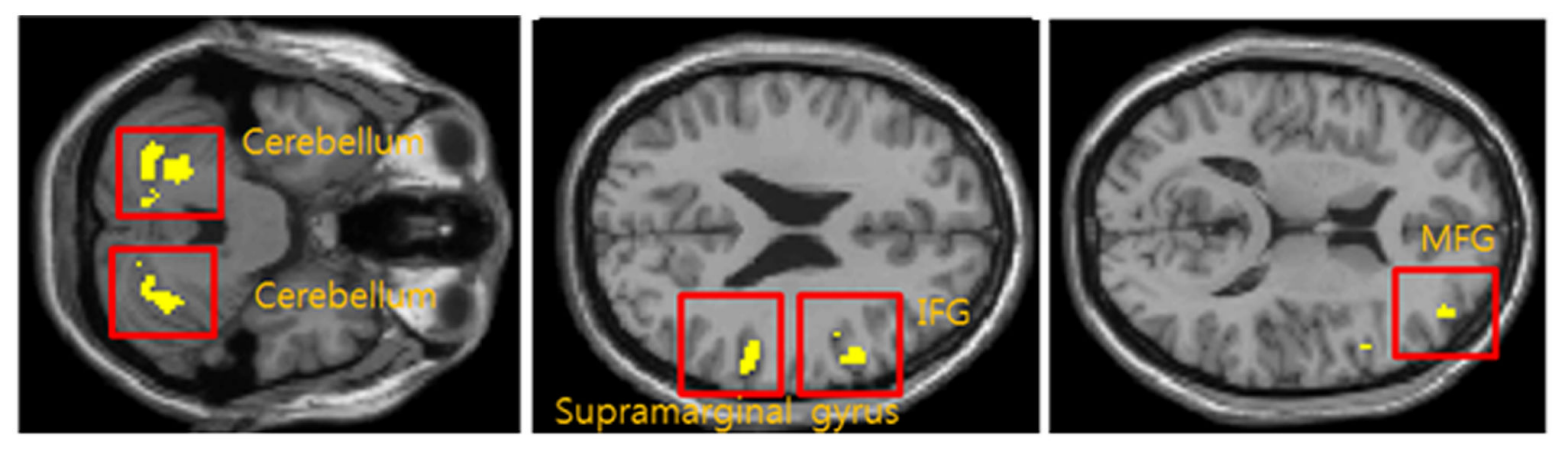
Commonly activated regions in FM patients and healthy control. Yellow clusters indicate common regions activated by painful stimuli in FM patients and healthy controls. Common regions include bilateral cerebellum, contralateral supramarginal gyrus, IFG and MFG.

The pressure-pain stimulation delivered to the healthy control group resulted in a significantly increased fMRI signal in nine brain regions (Table 1). The activated regions were the ambilateral supramarginal gyrus and cerebellum, contralateral superior frontal gyrus (SFG), inferior frontal gyrus (IFG), middle temporal gyrus (MTG) and thalamus, ipsilateral calcarine. Figure 4 shows activation regions common to both groups. Delivery of intolerable subjective pain to both groups resulted in brain activation in five common regions; these were the ambilateral cerebellum, and the contralateral gyrus, IFG, and medial frontal gyrus (MFG). fMRI signals in 13 regions of the brain in the patient group were more significantly augmented than in healthy controls: ambilateral cerebellum, MTG and MFG, contralateral supramarginal gyrus, IFG, putamen and insula, ipsilateral postcentral gyrus, IPL, and caudate (Table 2).

**Table 1:** Regions of statistically increased fMRI signal in healthy controls.

		**Contralateral**				**Ipsilateral**			
Cluster	Functional region	*x*	*y*	*z*	z-score	*x*	*y*	*z*	z-score
Supramarginal gyrus	SII	50	-3	26	5.07	-60	-32	32	3.55
SFG	SMA	28	58	8	4.78				
Cerebellum		30	-70	-20	4.26	-36	-62	-26	4.18
IFG		46	36	8	4.38				
Calcarine						-2	-72	10	4.18
MTG		40	48	16	4.03				
Thalamus		16	-12	12	3.58				

*p* < 0.001, FDR < 0.05.

SFG, superior frontal gyrus, IFG, inferior frontal gyrus, MTG, middle temporal gyrus.

**Table 2:** Regions of significantly increased fMRI signal in FM patients.

		**Contralateral**				**Ipsilateral**			
Cluster	Functional region	*x*	*y*	*z*	z-score	*x*	*y*	*z*	z-score
Supramarginal gyrus	**SII**	58	-28	20	4.96				
IFG		58	16	24	4.72				
Cerebellum		26	-72	-20	4.65	-28	-62	-26	4.70
MTG		60	-50	-2	4.61	-58	-52	4	4.03
MFG		38	2	52	4.32	-34	18	40	3.91
Postcentral gyrus	**SII**					-62	-22	22	4.15
IPL	**SI**					-46	-54	46	4.00
Putamen		28	4	-4	3.91				
Insula		38	-18	6	3.89				
Caudate						-16	-14	24	3.65

*p* < 0.001, FDR < 0.05

MFG, middle frontal gyrus, IPL, inferior parietal lobe

**Figure 5:**
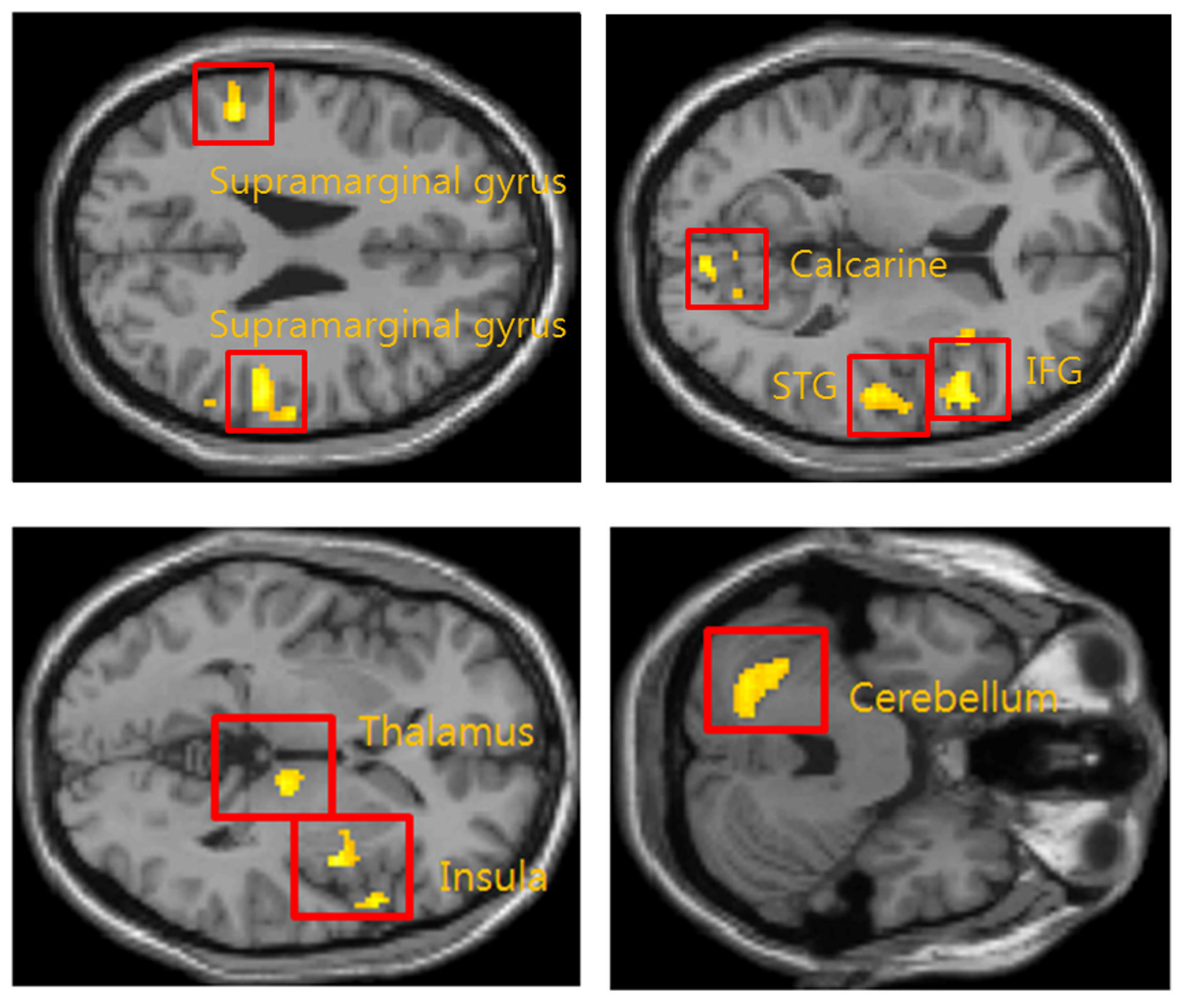
Comparison between FM patients and healthy controls. Augmented brain activation regions in FM patients compared with healthy controls resulting from the same level of subjective pain intensity (GBS level 14). Regions are Bilateral Supramarginal gyrus, ipsilateral cerebellum, contralateral calcarine, STG, IFG, thalamus and insula.

Table 3 and Figure 5 show the augmented brain activation regions in the FM patient group compared with the healthy controls for equal subjective pressure-pain intensity. Applying the same levels of subjective pain intensity (14 on GBS) resulted in more significantly increased BOLD signal than in the healthy controls in eight regions: bilateral supramarginal gyrus, and the contralateral insula, IFG, thalamus and calcarine, and the ipsilateral cerebellum and superior temporal gyrus (STG).

**Table 3:** Comparison of brain activation regions between FM patients and healthy controls.

		**Contralateral**				**Ipsilateral**			
Cluster	Functional region	*x*	*y*	*z*	z-score	*x*	*y*	*z*	z-score
Cerebellum						-34	-58	-24	4.96
Supramarginal gyrus	**SII**	56	-30	30	4.55	-52	-38	24	4.42
Insula		42	-2	2	4.24				
STG	**SII**					-40	-4	-12	4.21
IFG		52	10	8	4.06				
Thalamus		12	-20	2	3.99				
Calcarine		8	-84	8	3.98				

*p* < 0.001, FDR < 0.05

STG, superior temporal gyrus

### Comparison of brain activation pre- and post-treatment

**Figure 6:**
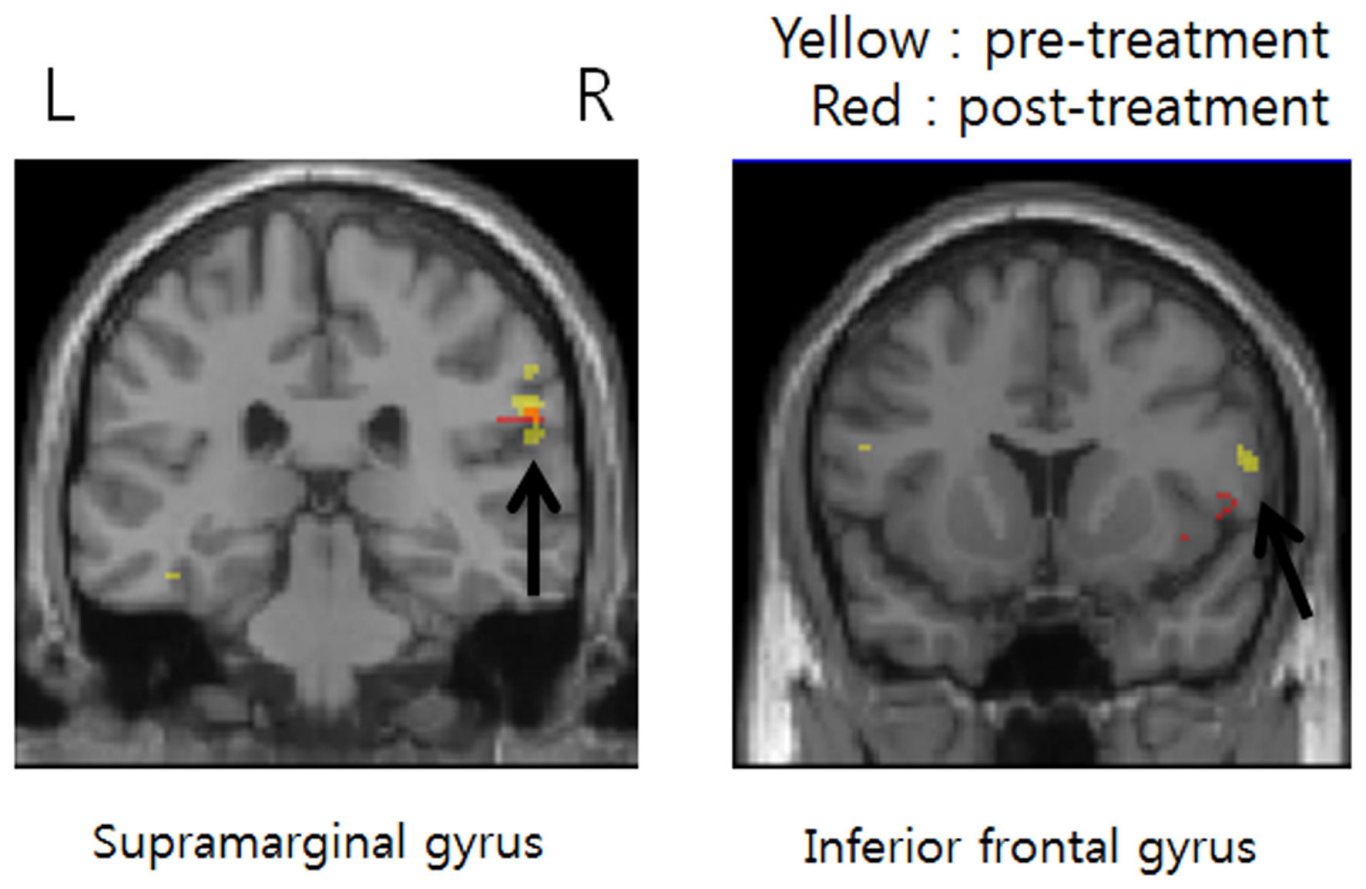
Commonly activated regions in pre-treatment and post-treatment. Functional magnetic resonance images showing activation in two regions, the supramarginal gyrus (arrow in left image) and inferior frontal gyrus (arrow in right image), during pressure-pain stimulation of subjectively strong intensity at both pre-treatment (yellow) and post-treatment (red) in the responder subgroup of FM patients.

fMRI data were acquired from seven female responder subjects (54.43±3.51 years) before and after pregabalin treatment (inter scan interval was 17.57±9.73 days; mean±SD). The pressure-pain stimulation delivered to the pre-treatment patients resulted in significantly increased BOLD signal in 13 brain regions (Table 4), and there were three significant activations post-treatment (Table 5). Figure 6 shows common activation (z-score) maps in the coronal image direction for subjectively strong pressure-pain stimulation pre- (yellow) and post-treatment (red). Two common brain regions, the supramarginal gyrus (arrow in left image) and inferior frontal gyrus (arrow in right image) exhibited increased fMRI signals.

**Table 4:** Regions of significantly increased fMRI signal in responders pre-treatment.

		**Contralateral**				**Ipsilateral**			
Cluster	Functional region	*x*	*y*	*z*	z-score	*x*	*y*	*z*	z-score
Supramarginal gyrus	**SII**	60	-36	28	4.83	-60	-34	34	3.45
IFG		44	8	24	4.48	-48	10	16	4.13
Putamen		28	2	-2	4.26				
Precentral gyrus	**MI**	40	-4	48	4.24				
Insula						-30	16	-6	3.98
MFG		36	36	42	3.90				
IPL	**SI**	50	-54	46	3.71				
Thalamus		14	-22	8	3.59	-10	-18	10	3.74
Cerebellum		28	-66	-20	3.61	-26	-64	-24	3.56

*p* < 0.001.

**Table 5:** Regions of significantly increased fMRI signals in responders post-treatment.

		**Contralateral**				**Ipsilateral**			
Cluster	Functional region	*x*	*y*	*z*	z-score	*x*	*y*	*z*	z-score
Supramarginal gyrus	**SII**	50	-30	26	3.59				
IFG		54	14	6	3.35				
Insula		42	14	-6	3.38				

*p* < 0.001.

**Figure 7:**
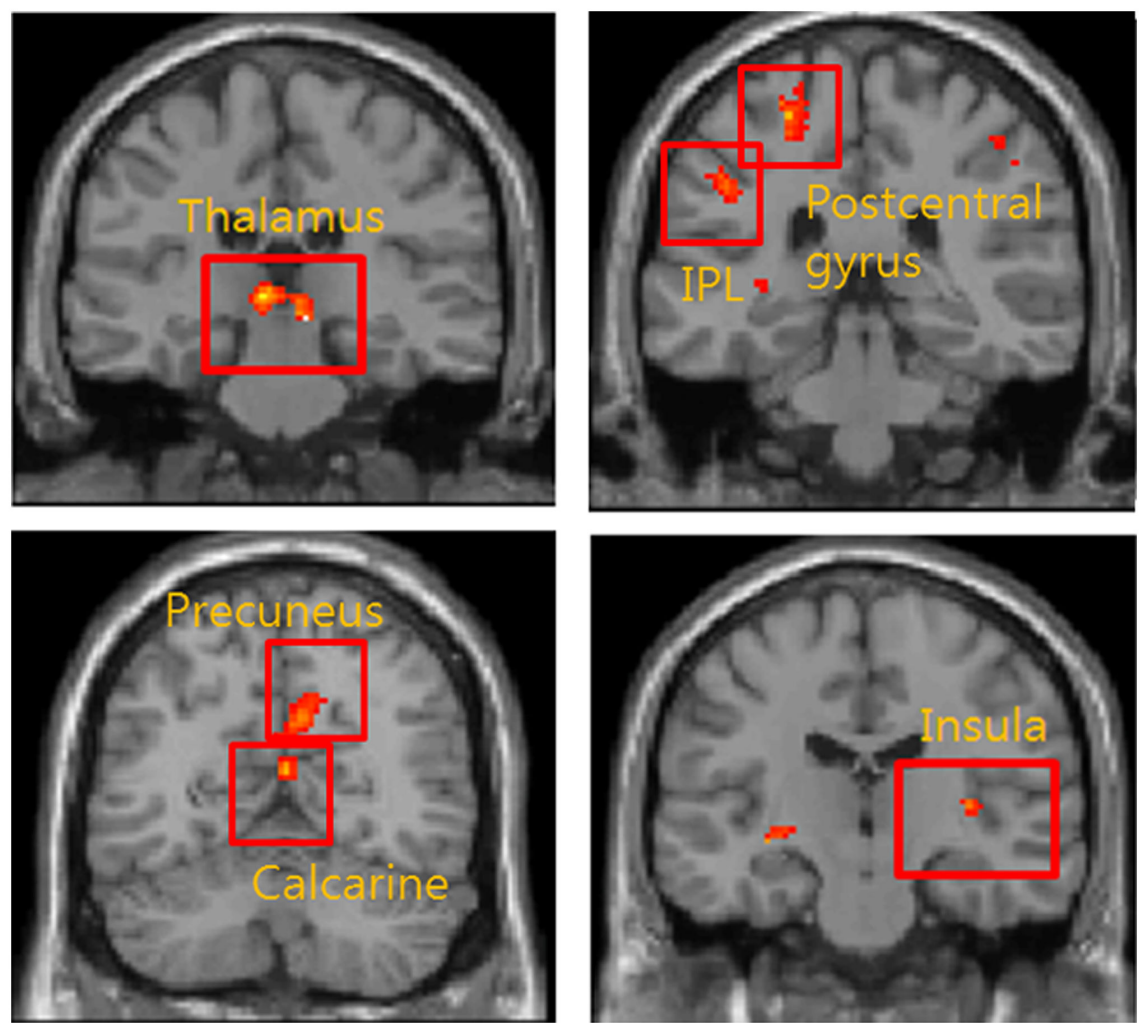
Comparison between pre-treatment and post-treatment. Coronal view of functional magnetic resonance images showing activation regions with significantly increased BOLD signals at pre- *versus* post-treatment in the responder subgroup of FM patients. In bilateral thalamus, IPL, contralateral precuneus, calcarine and ipsilateral insula, BOLD signal of pre-treatment was greater than post-treatment.

As shown in Table 6 and Figure 7, the fMRI signal pre-treatment was significantly greater than that post-treatment in 9 regions of the brain; ipsilateral thalamus, postcentral gyrus, inferior parietal lobule, contralateral thalamus, calcarine, middle frontal gyrus, middle cingulate cortex, precuneus, and insula.

**Table 6:** Significantly activated regions in responders post- *versus* pre-treatment.

		**Contralateral**				**Ipsilateral**			
Cluster	Functional region	*x*	*y*	*z*	z-score	*x*	*y*	*z*	z-score
Thalamus		6	26	-6	-3.54	-6	-26	2	-4.24
Postcentral gyrus	**SI**					-24	-32	60	-3.87
Calcarine		2	-60	14	-3.82				
MFG		36	34	44	-3.80				
MCC		12	24	40	-3.61				
IPL	**SI**	46	-36	50	-3.61	-44	-32	36	-3.43
Precuneus		6	-60	32	-3.57				
Insula		34	-18	6	-3.53				

*p* < 0.01.

MCC, middle cingulate cortex

### Comparison of brain activation responders and non-responders

**Figure 8:**
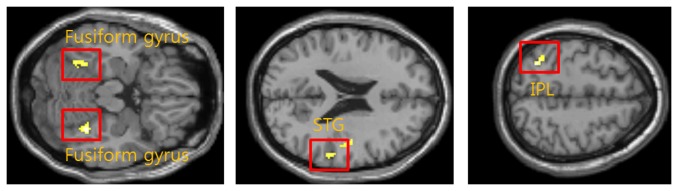
Comparison between responders and non-responders according to medication therapy. Regions including bilateral fusiform, ipsilateral IPL and contralateral STG were more activated in responders.

After acquiring baseline fMRI data, FM patients were divided into 9 responders (52.56 ± 3.89 years) and 10 non-responders (49.83 ± 10.10 years) by considering effect of medication therapy. And it was analyzed to compare difference of brain activation delivered painful stimulation between both groups. Intensity of stimulation was not significantly different between responders and non-responders. Table 7 and Figure 8 represent result of comparison of brain activation between responders and non-responders. Non-responder group was excepted 2 patients because one has drunk one glass of alcoholic drink and another’s fMRI data have been damaged. BOLD signal of responders was significantly greater than non-responder at 4 regions; bilateral fusiform gyrus, contralateral IPL and ipsilateral STG. 

**Table 7:** Comparison of brain activation regions between responders and non-responders.

		**Contralateral**				**Ipsilateral**			
Cluster	Functional region	*x*	*y*	*z*	z-score	*x*	*y*	*z*	z-score
Fusiform gyrus		36	-46	-18	3.08	-28	-46	-18	2.62
IPL	**SI**					-30	-60	54	2.96
STG	**SII**	58	-30	22	2.63				

*p* < 0.01.

## Discussion

The aim of this study was to evaluate alterations in neuronal activity during applied pressure-pain stimulation in FM patients following pharmacological treatment. Our findings demonstrated that abnormal neuronal activities were decreased following appropriate longitudinal drug therapy. These results support the hypothesis that FM is caused by alterations in neuronal pain processing and that pregabalin restricts the release of neurotransmitters in the pain-processing path.

The efficacy of pregabalin was evaluated in terms of decreasing pain and related clinical scores in FM patients [9,15,16]. The FM patient group had significantly lower threshold values of pain sensitivity than the healthy controls, but brain activation was evoked in patterns similar to those produced by comparable subjectively painful conditions. Under similar stimulus intensity conditions, FM patients felt as high pain as healthy controls [17–19]. Additionally, pain sensitivity in the FM patient group decreased post-treatment to approximately that of the healthy control group. Although clinical scores post-treatment in FM patients were not equal to those in the healthy controls, they were reduced by approximately half of the pre-treatment difference. According to Crofford et al. [9], pregabalin reduced pain scores and improved the sleep and fatigue significantly, improving the three major symptoms in patients with FM.

The pain threshold in FM patients was significantly lower than that of healthy controls, but brain activation evoked by the same subjective intolerable pressure pain in both groups was similar. These same subjective pain conditions resulted in the common brain activation areas in both groups: the bilateral cerebellum, contralateral supramarginal gyrus, IFG, and MFG. In contrast, the FM patient group showed significant activation in 13 areas but the healthy control group showed them in only nine areas. These results showed that FM patients were more sensitive than healthy controls to the same subjective pain condition. Gracely et al. [3] found that the brain activations evoked with similar stimulus pressures in FM patients and healthy control subject resulted in significantly different effects in the SI, IPL, insula, PCC, SII and cerebellum. These results are consistent with ours in the cerebellum, SII, and insula. Additionally, prediction of a painful stimulus has been shown to increase activity in SII [20], and increased activity in the insula cortex during the anticipation of pain [21]. Thus, this suggests that our results likely reflect abnormal pain processes in the SII and insula of FM patients. However, other activation regions must be considered because effects attributable to psychological factors, such as attention and anxiety, are also potentially powerful.

Pregabalin was used as the pharmacological therapy in this study. In the pre-treatment group, we observed brain activation in the SII, IFG, putamen, and cerebellum region (Table 4). In particular, activation of the SII, IFG, thalamus, and cerebellum was revealed on the both the contralateral and ipsilateral sides. This result is similar to the findings of another study that used mechanical stimulation [3]. These activations were more pronounced in the pre-treatment group, suggesting an augmentation of painful input to structures involved in processing the sensory discriminative components of pain. Both of pre- and post-treatments showed common significant increases in fMRI signals in two localized areas, the contralateral SII and IFG. A previous study reported that higher risk aversion was correlated with higher activity in the IFG [22]. As these activations increased in painful stimulation conditions, the authors assumed that the pain process was involved in the discriminative sensory and affective motivational components of pain.

In pre- versus post-treatment group, we found that pre-treatment shown greater activation than post-treatment at thalamus, postcentral gyurs, IPL, calcarine, MFG, MCC, precuenus including insula. According to another study, Koeppe and colleagues [23] reported that treatment of FM patients with the 5-HT_3_ receptor antagonist tropisetron reduced rCBF in the contralateral primary somatosensory (SI), posterior insula, and ACC. Their result is also consistent with our findings of a reduced fMRI signal evoked in the insula, thalamus and precuneus (somatosensory association cortex). Functional magnetic resonance imaging experiments have revealed that the insula plays an important role in acute experimental pain [24–26]. The insula does not simply process pain signals; the mid-posterior insula has also been implicated in awareness, and the middle cingulate cortex with cognitive processes, including attention to behaviorally relevant stimuli [27]. The reduced activity in the contralateral posterior insula and middle cingulate cortex suggests that the motivational affective component of pain was also affected by the treatment. The thalamus is generally considered to act as a relay between various subcortical areas and the cerebral cortex. Indeed, every sensory system includes a thalamic nucleus that receives sensory signals and sends them to the associated primary cortical area [28]. The reduced activations in the contralateral primary somatosensory cortex and thalamus post-treatment indicate altered processing of the sensory-discriminative dimension of pain. The posterior insula receives an input from spinothalamically activated ventral posterior inferior (VPI) thalamic nuclei [29]. Furthermore, according to Duncan et al., somatosensory thalamic stimulation activates the SI, SII, and insula [30]. From this point of view, activation of the thalamus is considered to be involved in both sensory-discriminative and affective motivational components of pain. The possibility should be considered that alterations in the patient’s reported evaluation of their pain, both sensory-discriminative and motivational-affective connectivity, might be changed between the thalamus and somatosensory cortex and posterior insula.

Pregabalin is known to effect primarily by modulation of calcium channel and it have been shown to be effective in neuropathic pain, but patients with chronic pain respond well to treatment and others show poor response [31–33]. Similarly, our study showed that FM patients were divided into 9 responders and 10 non-responders. We tried to observe difference of brain activation caused by painful stimulation between responders and non-responders. As a result, responders group shown that brain activation was greater than non-responders group at fusiform gyrus, IPL and STG. Therefore, The result suggests that regions mentioned above are relevant to respond for pregabalin, however, we have no explicit knowledge of the likelihood of response or non-response to pregabalin. Moreover, this result have a controversial point. This result was analysed using baseline fMRI data before pregabalin treatment. To suggest a reliable result, we think that it is necessary to approach by additional analysis after classifying responder and non-responder. This study has several limitations. First, it is lacking in terms of the number of patients who responded to PGB treatment. Second, we obtained no placebo-control fMRI data. However, our findings indicate that PGB treatment improved the responder’s brain activation in pressure-pain stimulation. Thus, it can be viewed as a primary study of FM patients and pharmacological therapy and could be used to decide whether a placebo-controlled experiment is needed. Further research on neuro-imaging after PGB treatment would help clarify the association between brain activation and pain sensation in FM patients.

In conclusion, our findings suggest that pregabalin has an influence on aspects of the whole pain matrix. This method can be applied to longitudinal clinical trials of other pharmacological therapies for FM. Longitudinal changes in brain connectivity resulting from drug therapy will be included in a future study, which will clarify the mechanisms of the pain matrix. 
